# Long-lasting antiviral innate immune priming in the Lophotrochozoan Pacific oyster, *Crassostrea gigas*

**DOI:** 10.1038/s41598-017-13564-0

**Published:** 2017-10-13

**Authors:** Maxime Lafont, Bruno Petton, Agnès Vergnes, Marianna Pauletto, Amélie Segarra, Benjamin Gourbal, Caroline Montagnani

**Affiliations:** 1 Ifremer, IHPE, UMR 5244, Univ. Perpignan Via Domitia, CNRS, Univ. Montpellier, F-34095 Montpellier, France; 2 Univ. Perpignan Via Domitia, IHPE UMR 5244, CNRS, IFREMER, Univ. Montpellier, F-66860 Perpignan, France; 3 Ifremer, LEMAR UMR6539, F-29840 Argenton-en-Landunvez, France; 4 Department of Comparative Biomedicine and Food Science. University of Padova, Viale dell’Università 16, 35020 Legnaro (PD), Italy; 5 Univ. Brest Occidentale, LEMAR UMR 6539 CNRS/UBO/IRD/Ifremer, Institut Universitaire Européen de la Mer, F-29280 Plouzané, France

## Abstract

In the last decade, a paradigm shift has emerged in comparative immunology. Invertebrates can no longer be considered to be devoid of specific recognition and immune memory. However, we still lack a comprehensive view of these phenomena and their molecular mechanisms across phyla, especially in terms of duration, specificity, and efficiency in a natural context. In this study, we focused on a Lophotrochozoan/virus interaction, as antiviral priming is mostly overlooked in molluscs. Juvenile *Crassostrea gigas* oysters experience reoccurring mass mortalities events from Ostreid herpes virus 1 with no existing therapeutic treatment. Our results showed that various nucleic acid injections can prime oysters to trigger an antiviral state ultimately protecting them against a subsequent viral infection. Focusing on poly(I:C) as elicitor, we evidenced that it protected from an environmental infection, by mitigating viral replication. That protection seemed to induce a specific antiviral response as poly(I:C) fails to protect against a pathogenic bacteria. Finally, we showed that this phenomenon was long-lasting, persisting for at least 5 months thus suggesting for the first time the existence of innate immune memory in this invertebrate species. This study strengthens the emerging hypotheses about the broad conservation of innate immune priming and memory mechanisms in Lophotrochozoans.

## Introduction

In the last decade, a radical paradigm shift has emerged in immunity. It is now clear that the innate immune system is capable to provide forms of immune memory as demonstrated by the compelling studies emerging on invertebrates, plants, vertebrates, and even bacteria^1^. These studies have shown in a wide range of organisms lacking conventional adaptive immune responses that they can adapt upon primary exposure and are capable to implement an improved immune response upon secondary exposure to a parasite (including macro- and micro-parasites as bacteria and viruses)^2–8^. In vertebrates, seminal papers from Netea *et al*. showed that cells of the innate immune system can also build immunological memory notably *via* epigenetic reprogramming allowing for enhanced non-specific inflammatory responses upon secondary challenges^9^. Since these processes rely on different molecular mechanisms than those of adaptive immunity (Ig- and lymphocyte-mediated), they have been designated as immune priming or innate immune memory in invertebrates, and trained immunity in vertebrates^10^. Those data have blurred the boundaries between adaptive and innate immune responses that have even been described as a continuum of host defences and they currently challenge the commonly accepted dogmas in immunity. Since this field of research is still in its infancy, it is very delicate to come up with a universal description of innate immune priming. The broadest definition of these processes would be the ability to store or re-use the information on a previously encountered non-self antigen or parasite, to induce enhanced resistance or tolerance (in terms of immune response, parasite elimination and host survival capacities) upon secondary exposure.

Studies testing this phenomenon in invertebrates appear to be quite heterogeneous and largely differ in terms of experimental design, host-parasite combinations, elicitors used for priming (pathogenic *versus* inactivated pathogens or non-infectious agents), time span between priming and challenge, route of priming (oral *versus* injection) and the degree of the demonstrated specificity of the primed response^11,12^. Moreover this improved protection has been observed either within the same developmental stage (within generation immune priming), across life stages (ontogenic immune priming), or across generations (transgenerational immune priming). Regarding the time span between priming and challenge, it can extend for weeks, sometimes for almost the lifetime of the organism (from larval stage to adult), but it remains in most cases rather short and does not extend over 3 months^11^. However, those studies have mainly focused on arthropods regarding bacteria and macro-parasite interactions. To date, antiviral immune priming has been explored in a handful studies on *Caenorhabditis elegans* and insects^13,14^. Most studies were focusing on crustaceans where several studies reported the potential development of vaccines to combat a major viral pathogen (the white spot syndrome virus, see ref.^15^ for review). Hence available evidence for innate immune priming is still fragmentary and we still lack a complete view of this phenomenon across phyla especially in terms of duration and specificity, and efficiency in a natural context and underlying molecular mechanisms.

In Lophotrochzoans, studies conducted over the last ten years on the gastropod *Biomphalaria glabrata* has provided compelling evidence of innate immune memory against the parasite *Schistosoma mansoni*. *B*. *glabrata* is able to mount a highly specific, inducible and time-dependent protection against different species or strains of *Schistosoma* in a genotype-dependant manner^7,16^. Immune priming seems to be supported by snail humoral factors that lead to the degeneration and death of the parasite^7^ following a shift from cellular to humoral response upon secondary infection^17^. In the present study, we focused on a marine bivalve mollusc, the Pacific oyster *Crassostrea gigas* (now termed *Magallana gigas*, Thunberg, 1793)^18^. *C*. *gigas* is the main aquaculture animal species produced in the world, exploited in more than 27 countries (FAO Fisheries & aquaculture). But this species has been subjected to recurrent mortality events plaguing oyster production worldwide. Since 2008, a pathogen has been strongly associated to mortality events impacting juvenile oysters, the herpes-like virus OsHV-1 µvar (Ostreid herpes virus 1) that can induce a 100% mortality rate^19–21^. In bivalves information are still scarce and to date only a few studies have investigated innate immune priming mechanisms. In the scallop *Chlamys farreri*, authors showed an improved immune response and survival to the bacterium *Vibrio anguillarum* following previous exposure to the same pathogen. In *C*. *gigas*, sceptic immune priming with heat killed *Vibrio splendidus* conferred enhanced phagocytic responses and haematopoiesis upon re-infection with the same pathogen^22,23^. In a previous work, we developed a model system for oyster/virus interaction using a non-infectious elicitor, the viral mimic synthetic double stranded RNA (dsRNA) called poly(I:C). Poly(I:C) has been known to be a key signature of viral infection and widely used as a viral mimic in vertebrates and has been used in fish models to enhance antiviral defenses^24–27^. As a non-hazardous synthetic compound, it also ensures good reproducibility between experiments and safety. Importantly, dsRNA is also produced by DNA viruses such as herpes viruses, likely due to overlapping converging transcription of viral genes^28^. Recent data also tested the impact of prior injection of dsRNA on viral protection^29^. These studies showed that short-term priming with poly(I:C) and dsRNA injections were able to develop an antiviral state mitigating the viral loads in oysters upon subsequent viral challenge and inducing genes from an IFN-like pathway^29–31^. Recent data also suggested that this effect could be maintained across generations^32^.

These researches have opened up new perspectives to explore immune defenses of these phylogenetically key and commercially important animals. Understanding this aspect of immunology in bivalves is of main importance in light of the global concern of increasing epizootic disease outbreaks currently affecting oysters. But as of yet, we still lack a comprehensive view of the antiviral immune priming phenomenon in the oyster. In particular, it is unknown whether the different dsRNA or other nucleic acids are equally efficient to prime oysters; if poly(I:C) is able to reduce mortalities as well as viral loads; if the protection can extend over 54 h of priming; if that protection is restricted to antiviral response and if it is efficient in the environment. To deeply characterize this phenomenon, we present here a comprehensive phenomenological approach of antiviral immune priming processes in the oyster. We explored the protective effect of nucleic acid exposition in comparing various types of nucleic acids (dsRNA, ssRNA, various lengths, synthetic) and testing priming efficiency against a bacterial as well as lasting effect by following survival and pathogen clearance throughout the experiments. Furthermore, we validated these processes in the field in looking at host survival during a naturally occurring disease outbreak.

## Results

### Various nucleic acid injections enhance oyster survival upon viral infection

**Figure 1:**
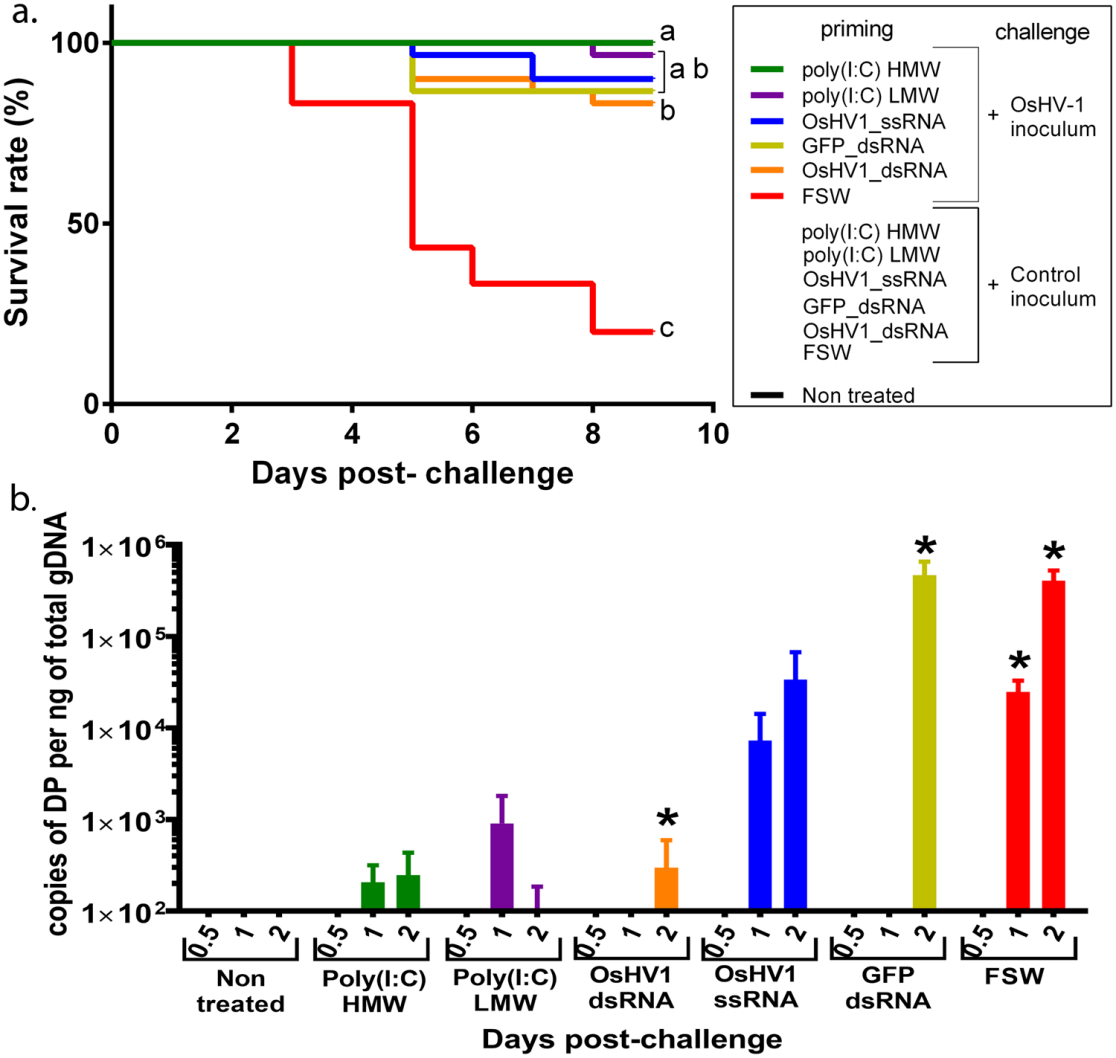
Poly(I:C) as other dsRNA can protect against viral infection and reduce mortality due to viral infection (**a**) Kaplan–Meier survival curves were generated from spats primed by injection after anesthesia with 5 µg/animal of different nucleic acids (poly(I:C) HMW; poly(I:C) LMW; OsHV1_dsRNA; OsHV1_ssRNA; GFP_dsRNA) or filtered seawater (FSW) as a control and injected 1 day post-priming with OsHV-1 µvar homogenate (1.79 × 10^7^ copies of DNA polymerase -DP- gene per oyster) or OsHV-1-free control homogenate. Mortalities in each group of 30 oysters (10 per tank, n = 3 × 10 per treatment) were monitored for 9 days after infection. Controls reaching 100% survival appear hidden and merged behind the poly(I:C) primed line (green). Different letters next to the graphed lines indicate statistically significant difference among treatment at a-b, *p*-value < 0.05; b-c *p*-value < 0.0001 (log-rank test, n = 30). (**b**) OsHV-1 DNA detection by quantitative PCR in 3 pools of 3 spats in the same experiment. Results are expressed as the mean number of DP gene copies detected per ng of genomic DNA extracted from whole spat pools. The effect of priming on OsHV-1 loads was determined using Kruskal-Wallis test (*p-value* < 0.05). Significant differences between treatments were performed compared to T0 using Mann-Whitney (n = 3 pools of 3 oysters)(*p- value < 0.1).

Previous studies showed that HMW poly(I:C) and dsRNA were able to significantly reduce viral loads upon OsHV-1 infection but also mortality rates regarding dsRNA priming. Building on these observations, we compared the efficiency of the nucleic acids previously tested added to previously untested nucleic acids as LMW poly (I:C), OsHV1-dsRNA, OsHV1- ssRNA^29,30^. We aimed at assessing whether this phenomenon was specific to the dsRNA analog used. To test this, we primed oysters by injection with different types of nucleic acids 1 day before an OsHV-1 challenge. We tested the poly(I:C) HMW previously used, poly(I:C) of low molecular weight (LMW), in-house synthesized double- or single-stranded RNA derived from the gene encoding the green fluorescent protein (GFP) or from the ORF 87 of the OsHV-1 encoding an inhibitor of apoptosis. Survival rates after OsHV-1 challenge showed that all nucleic acids injected were able to significantly reduce oyster mortalities as opposed to the injection of the filtered seawater control that only reached 20% survival (80% mortality) (Fig. 1a). Poly(I:C) HMW and LMW were the most efficient leading to 100 and 97% survival, respectively, whereas GFP-dsRNA, OsHV1-dsRNA, and OSHV1-ssRNA lead from 83% to 90% survival. Altogether the prior injection of any nucleic acid 24 h before an OsHV-1 challenge significantly increased the survival rate of the oysters by at least 63%. As a first attempt to understand the mechanisms underlying this phenomenon, we quantified by quantitative PCR (qPCR) the OSHV-1 loads in oysters following challenge (Fig. 1b). Results indicated that oysters from the control group, FSW primed oysters, showed a high level of virus DNA loads with a peak at 4.10^5^ copies of DP (OsHV-1 DNA polymerase gene) per ng of oyster genomic DNA at 2 days post-infection. Regarding nucleic acid-treated oysters, poly(I:C) HMW and LMW showed no quantifiable amounts of viral DNA (below qPCR detection limit). The GFP-dsRNA-treated oysters showed significant amounts of virus DNA with viral loads reaching 4,6.10^5^ at 2 days post-infection. OsHV1-ssRNA and OsHV1-dsRNA treated oysters showed increasing viral loads reaching 3.10^4^ and 3.10^2^ copies of DP.ng^−1^ oyster DNA at 2 days post-infection, respectively. Altogether, our results revealed that the virus protection observed in the oyster against OsHV-1 wasn’t specific of a particular dsRNA. These nucleic acids cannot only reduce viral loads in OsHV-1 infected oysters but also reduce mortalities. Since the stronger responses were observed when priming with poly(I:C) HMW, we used it as a model to mimic DNA virus infection and further characterized antiviral protection and immune priming in oysters.

### Poly(I:C) treatment fails to protect against a pathogenic bacteria

**Figure 2:**
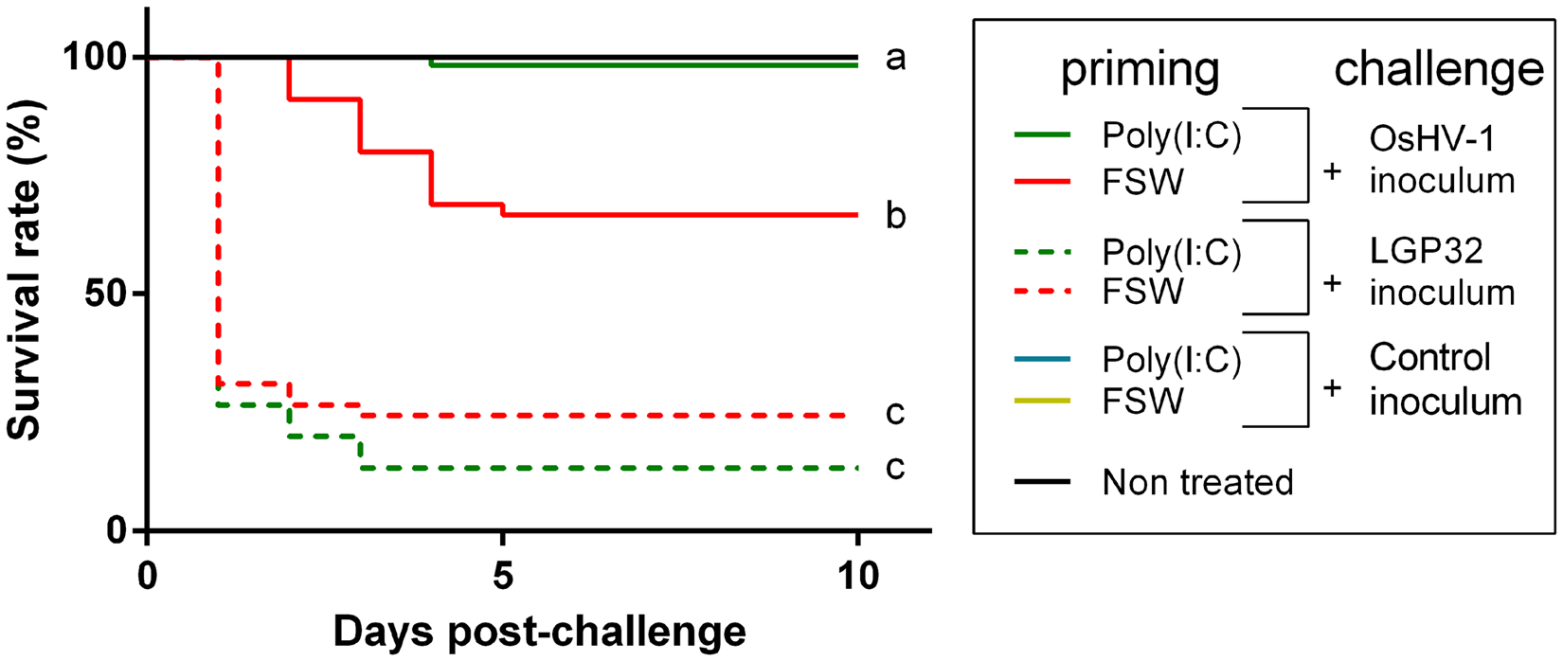
Poly(I:C) specifically protects against viral but not bacterial infection. Kaplan–Meier survival curves were generated from spats primed by injection after anesthesia with poly(I:C) (19 µg.g^−1^ of oyster) or with filtered seawater as a control and injected 1 day post-priming with OsHV-1 µvar homogenate (1.1 × 10^7^ copies of DP gene per oyster) or OsHV-1-free control homogenate or *Vibrio tasmaniensis* LGP32 (7 × 10^7^ UFC per oyster). One control conditions without injection is represented as non-treated (black line). Controls reaching 100% survival (poly(I:C) and FSW primed oysters challenged with the control inoculum) appear hidden and merged behind the non-treated control line. Mortalities in each group of 45 oysters (15 per tank) were monitored for 10 days after infection. a-b and b-c, *p*-value < 0.0001; log-rank test (n = 45).

Next, we tested whether this protection was specific to a viral infection. In a previous study we showed that injection of heat-killed bacteria did not protect against a subsequent viral infection suggesting that priming could be specific to virus protection^30^. To test this hypothesis, we performed the reverse experiment that is to say, priming with poly(I:C) before challenge with bacteria. But firstly, we tested the dose-dependency of poly(I:C) protection and established an optimal dose in monitoring protection efficiency from 1.9 ng to 19 µg of poly(I:C) per gram of oyster flesh (Supplementary Figure 1S). We showed that the protection efficiency was dependent on the quantity of poly(I:C) injected and was effective from a concentration of 0.019 µg.g^−1^ of oysters. We then used the maximal dose leading to the maximal survival rates (19 µg.g^−1^ of oysters) in all the experiments conducted herein. We primed oysters by injection with poly(I:C), or FSW as a control, 24 h before challenging them either with an OsHV-1 inoculum or a control inoculum prepared from virus-free oysters or a lethal dose of the pathogenic *Vibrio tasmaniensis* LGP32 bacterial strain (Fig. 2) ^33^. Results confirmed that poly(I:C) was efficient in protecting oyster against an OsHV-1 challenge with a 98% survival rate as opposed to FSW-challenged oysters demonstrating 74% survival. However, poly(I:C) failed to protect oysters against a bacterial infection demonstrating only 13% survival which was not significantly different from the FSW-primed oysters challenged by bacteria (24% survival). Altogether these results showed that poly(I:C) priming failed to protect against that one specific bacterial strain and might suggest that poly(I:C) specifically triggers antiviral response and protection.

### Poly(I:C) treatment triggers a long lasting antiviral protection also efficient during a naturally occurring disease outbreak

**Figure 3:**
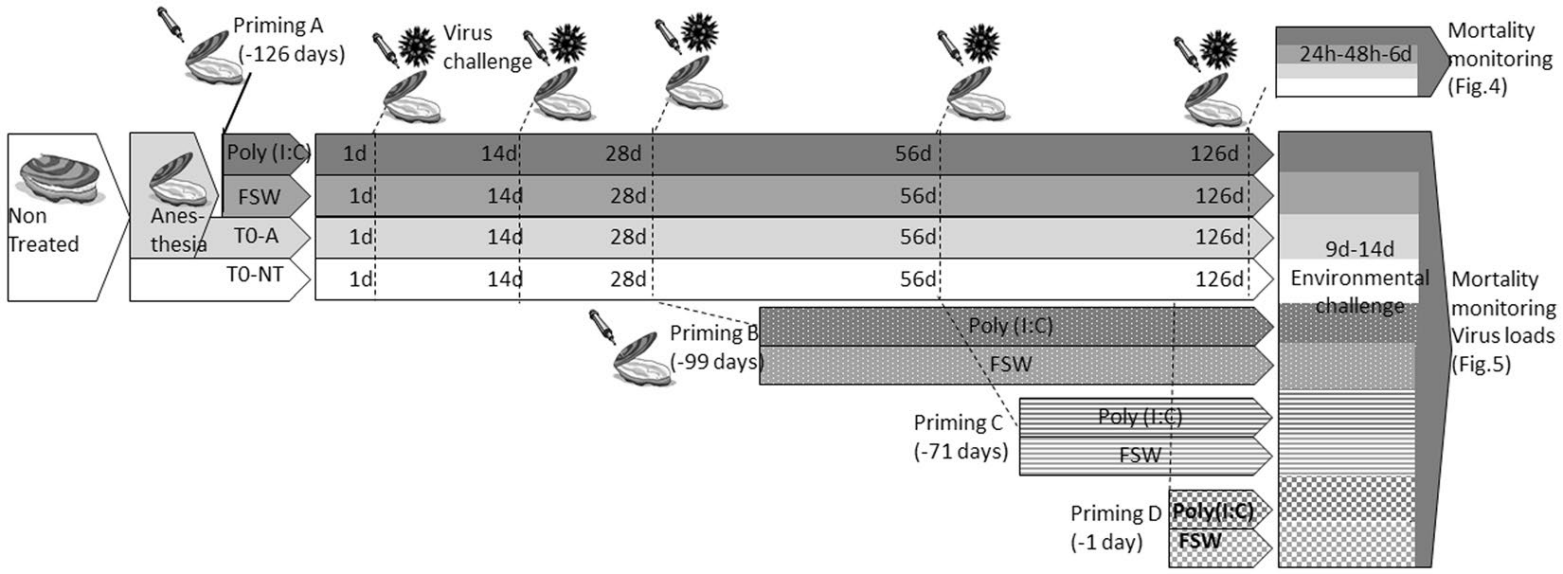
Schematic representation of the experimental design used to test the duration of poly(I:C) priming and the efficiency in the environment. The same batch of oysters was anaesthetized before being injected with poly(I:C) or filtered seawater (FSW). Four priming experiments were performed at different times: at 126 days (priming A), 99 days (priming B), 71 days (priming C), 24 h (priming D) before immersion in the Thau lagoon (Mediterranean Sea). For priming A, the antiviral effect of the treatment was tested at 1 day, 14 days, 28 days, 56 days or 126 days post-treatment in experimental infections with OsHV-1. Throughout all the experiments, a batch of oysters remained untreated as a control. During each experimental infection, mortalities were monitored daily post-challenge. During the environmental test, mortalities were monitored daily and sampling performed when mortalities occurred (9 and 14 days post-immersion).

**Figure 4:**
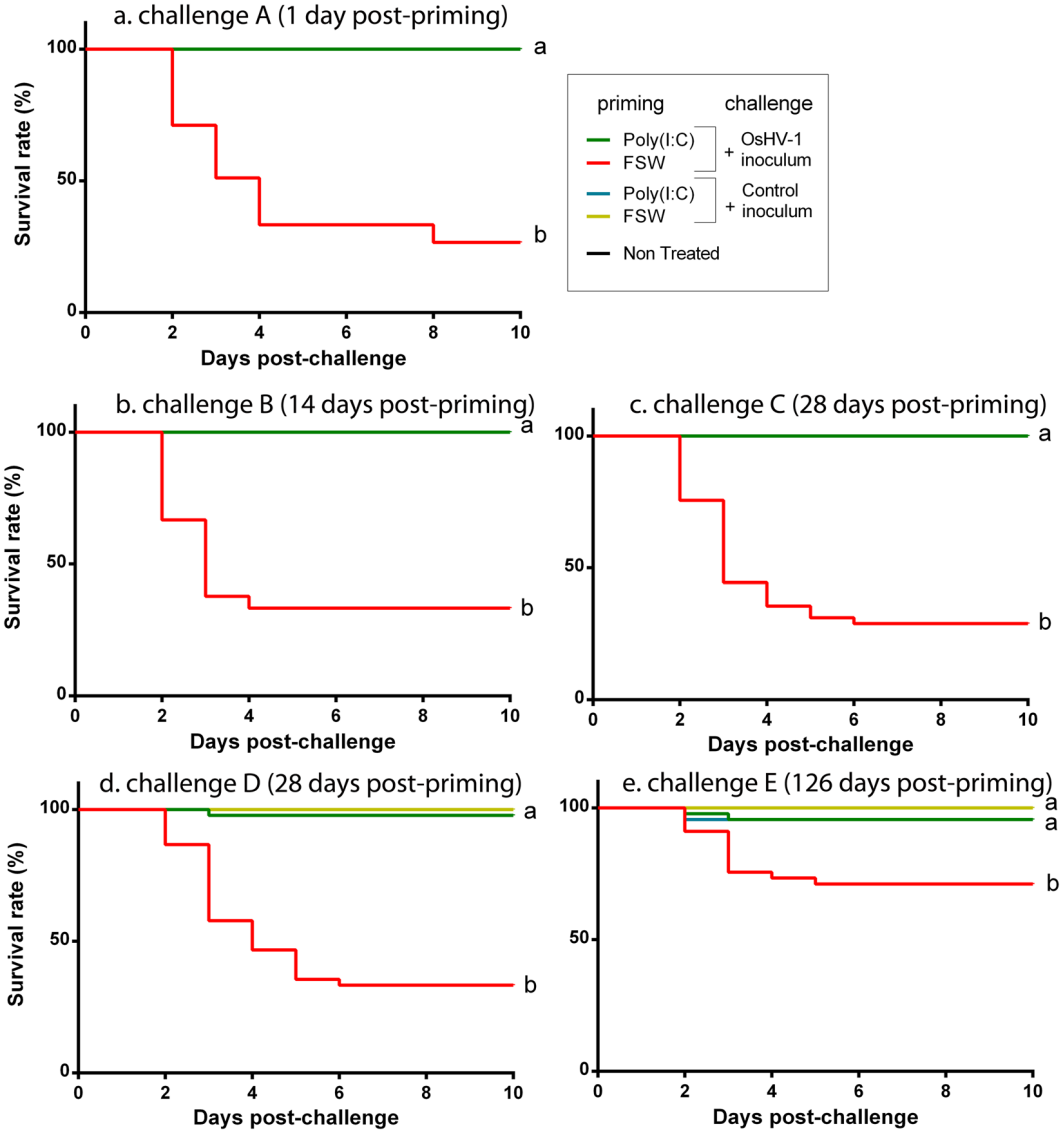
Duration of poly(I:C) priming. Kaplan–Meier survival curves were generated from spats primed by injection after anesthesia with poly(I:C) (19 µg.g^−1^ of oyster) or with filtered seawater as control and injected 1 day (**a**), 14 days (**b**), 28 days (**c**), 56 days (**d**) or 126 days (**e**) post-priming A with OsHV-1 µvar homogenate (7.7 × 10^6^ and 4.4 × 10^6^ copies of DP gene per oyster, respectively) or a pathogen free control homogenate. One control conditions without injection is represented as non-treated (black line). Conditions demonstrating 100% survival rates can appear hidden and merged with other ones reaching the same survival rate. Mortalities in each group of 45 oysters (15 per tank) were monitored for 10 days after infection. a, b indicate *p*-value < 0.0001; log-rank test (n = 45).

In the previous experiments presented here, antiviral protection has been observed only 1 day after poly(I:C) injection. To test whether this observed protection could be long-lasting, we expanded the lapse time between prior poly(I:C) priming and OsHV-1 challenge. To this end, oyster spats were primed by injection either with poly(I:C) or FSW as a control and stored in a bio-secured nursery facility. To determine if the poly(I:C) was still protecting from OsHV-1 infection, oysters were regularly challenged by injection with an OsHV-1 inoculum prepared from the same batch of infected oysters, at 1, 14, 28, 56 and 126 days after poly(I:C) treatment (see experimental scheme in Fig. 3). Kaplan-Meier survival curves at all time points, showed that poly(I:C) was still efficient in protecting oysters from 24 h to 126 days post-priming with consistent survival rates ranging from 89% to 96% (Fig. 4).

**Figure 5:**
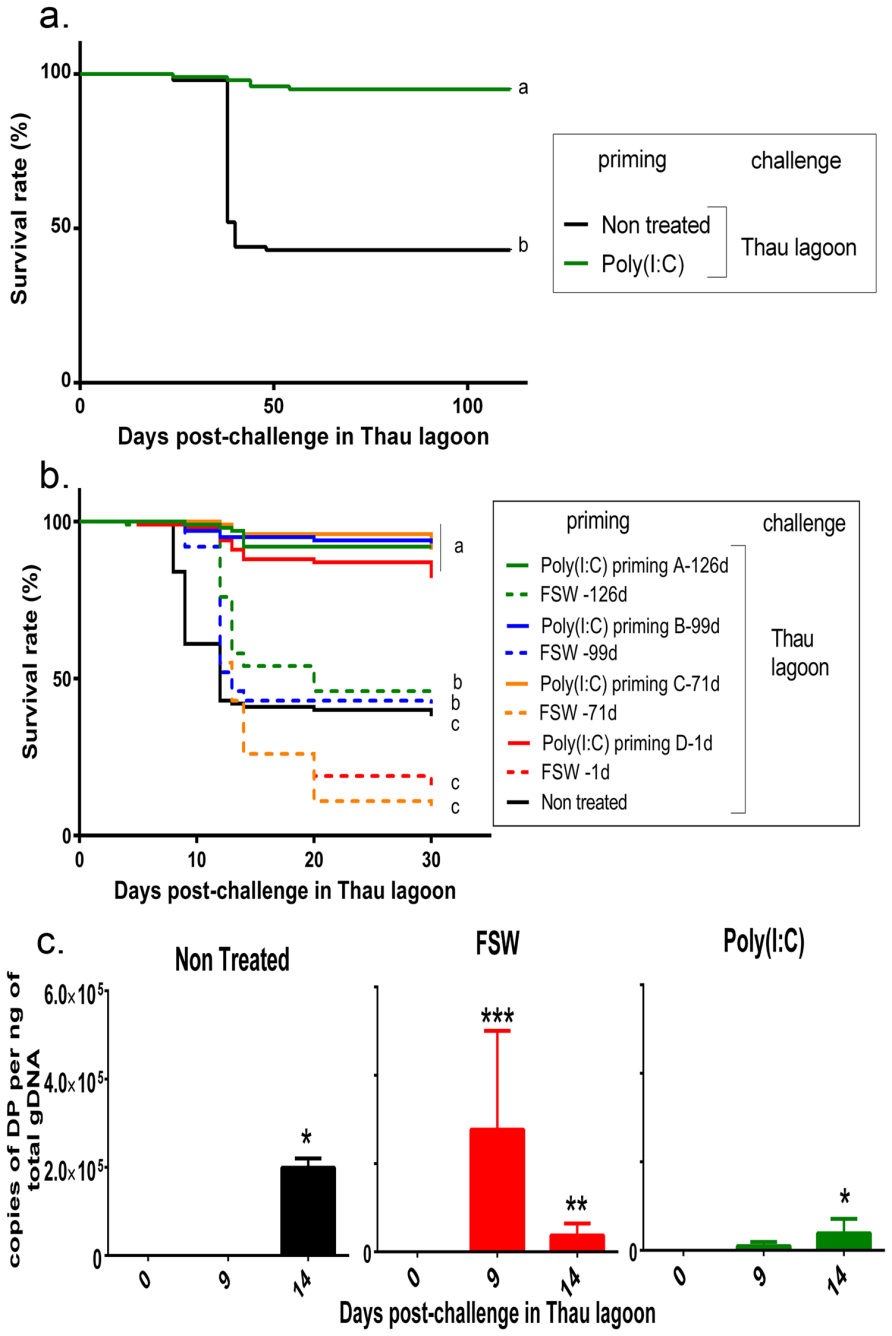
Poly(I:C) priming efficiency during a naturally occurring disease outbreak. (**a**) Kaplan–Meier survival curves were generated from spats primed by injection after anesthesia with 10 µg of poly(I:C) per oyster (green line) 1 day before deployment in a coastal lagoon of the Mediterranean sea (Thau Lagoon, France) before a disease outbreak (April 2015). Animals without treatment are represented as non-treated control (black line). Mortalities in groups of 100 oysters were monitored for 119 days after deployment. Different letters next to the graphed lines indicate statistically significant difference among treatment at *p*-value < 0.0001(log-rank test; n = 100). (**b**) Kaplan–Meier survival curves were generated from spats primed after anesthesia with poly(I:C) (19 µg.g^−1^ of oyster) (green lines) or with filtered seawater as a control (FSW, red lines), 126 days (priming A), 99 days (priming B), 71 days (priming C) or 1 day (priming D) before deployment in the Thau Lagoon during a disease outbreak (June 2016). Animals without treatment are represented as non-treated control (black line). Mortalities in groups of 100 oysters were monitored for 30 days after deployment. Different letters next to the graphed lines indicate statistically significant difference among treatment at a-b *p*-value < 0.0001; b-c p-value < 0.05 (log-rank test, n = 100). (**c**) OsHV-1 DNA detection on 2016 experiment, by quantitative PCR. Results are expressed as the mean number of DP copies detected per ng of genomic DNA extracted from whole spat pools at 9 and 14 days post immersion. The effect of priming on OsHV-1 μvar loads was determined using Kruskal-Wallis test. Statistical differences represent an increase of OsHV-1 loads compared to the loads at the beginning of the experiment (T0). Significant differences between treatments were performed using Mann-Whitney test (*p-value < 0.1; **p-value < 0.05; ***p-value < 0.01- n = 12 pools of 3 oysters for poly(I:C) and FSW treated oysters and n = 3 pools of 3 oysters for non-treated oysters).

**Figure 6:**
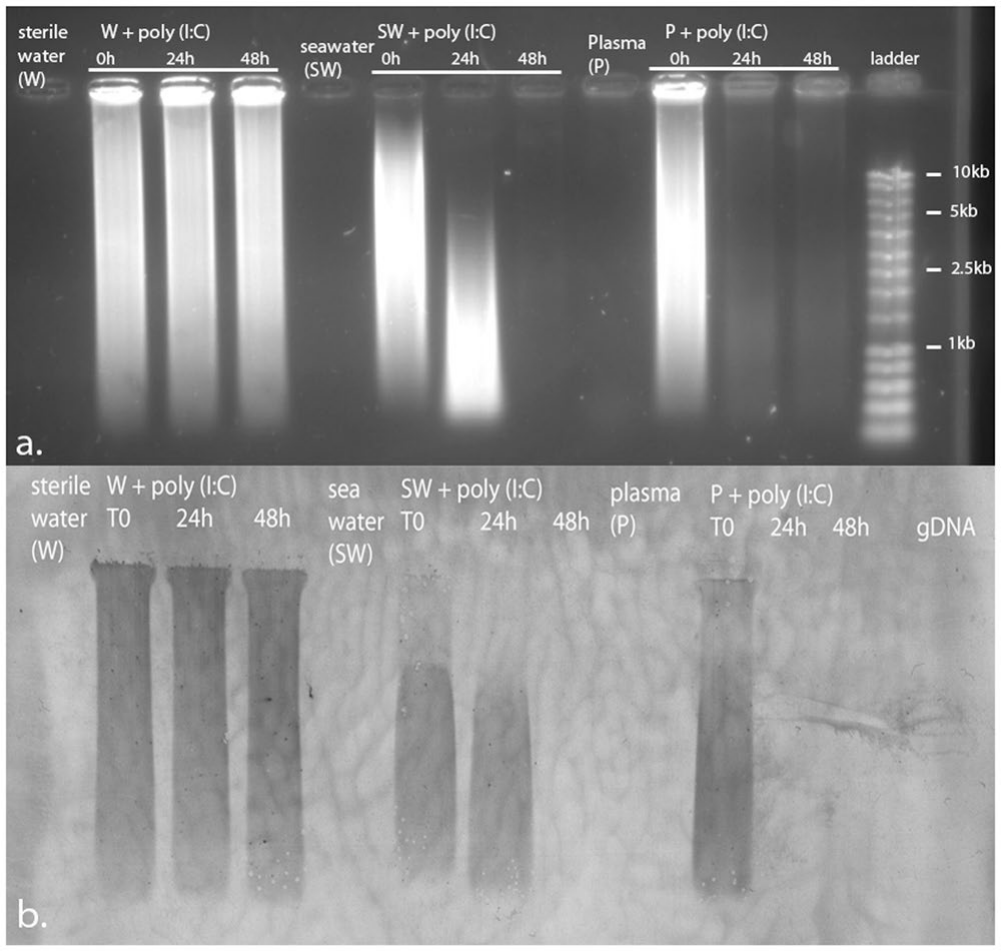
Poly(I:C) persistence in seawater and oyster hemolymph. Immuno-northern blotting using antibodies against double stranded RNA. Poly(I:C) was incubated for 0, 24 h, 48 h with sterile water (W), seawater (SW) or oyster plasma (P) (cell free hemolymph) and 1 µg of each sample separated in 1% agarose non-denaturing gel. A SmartLadder (MW-1700-02, Eurogentec) was used. Sterile water, seawater and plasma without addition of poly(I:C) were used as negative controls. After electrophoresis, (**a**) nucleic acids were stained using GelRed (image partially cropped) or (**b**) blotted onto a nylon membrane and immuno-localized using the J2 antibody (Scicons) (image partially cropped). For immune-blotting, 1 µg of *C*. *gigas* genomic DNA (gDNA) was added to the gel in place of the ladder, as a negative control. Full-length blots/gels are presented in Supplementary Figure 2S.

We then tested the efficiency of the poly(I:C) treatment in the Thau lagoon (Mediterranean Sea, France) affected by recurrent seasonal mortality events associated with OsHV-1. We deployed two distinct oyster cohorts twice in Thau lagoon in 2015 and 2016. In the first deployment, a first cohort of oysters were injected with poly(I:C) or non-treated as a control and then placed in the field before the disease outbreak (1 day after priming). Oysters primed with poly(I:C) had a 80% survival compared to control oysters which had 29% survival. Poly(I:C)-oysters had a 51% higher survival (Fig. 5a). Since mortalities occurred only 30 days after immersion in the field and that survival rates were maintained for 119 days after immersion, it suggested that priming duration could be extending largely over 1 day and could be long-term. To confirm this efficiency in the field and the long-lasting effect of poly(I:C), we designed a second experiment. As a mirror to the duration experiment, we primed oysters at different lapse times before deploying them in the Thau lagoon on a second year (2016) (Fig. 2). Using another cohort of specific pathogen-free oysters, we primed oysters by injection of poly(I:C) or filtered seawater as a control at 126 days, 99 days, 71 days and 1 day before challenge in the field (Fig. 5). Mortality monitoring showed that poly(I:C) was still very efficient in protecting oysters against a natural infection with 83% to 92% survival (Fig. 5b). The protection lasted until the end of the experiment, *i*.*e*. 156 days, more than 5 months after poly(I:C) initial exposition. In addition, in that second experiment, we quantified viral loads in oysters during the mortality outbreak (Fig. 5c). Results show that viral loads were high in non-treated oysters reaching 2.10^5^ copies of DP and FSW-treated oysters reaching a mean of 3.10^5^ copies of DP gene.ng^−1^ of oyster gDNA. In contrast, poly(I:C)-treated oysters showed reduced viral loads however reaching a mean of 3.10^4^ copies of DP.ng^−1^ at 14 days post-immersion. Nonetheless, those results confirmed that poly(I:C) is able to trigger a long-lasting antiviral state in the environment enabling oysters to resist to the disease. The maintenance of this protection over time could either be a direct consequence of the early poly(I:C) treatment but also to the persisting presence of poly(I:C) in oysters. To address that issue, we tested poly(I:C) stability in seawater and in the oyster hemolymph (circulating fluid or blood equivalent, cell free fraction) by following its presence using the J2 antibody raised against dsRNA (Fig. 6) ^28^. Poly(I:C) appeared as a smear as its size ranges from 1.5 kb to 8 kb. The J2 antibody allowed us to show that the smears observed were only due to the presence of poly(I:C), as negative controls (fluids without poly (I:C)) and gDNA did not induce any signal. No signals were obtained when blotting without the primary antibody (Supplementary Figure 2S). If poly(I:C) seemed stable in sterile water for 2 days, results showed a rapid degradation of poly(I:C) in seawater as well as in the hemolymph with a signal totally extinguished between 24 h (hemolymph) and 48 h (seawater) after contact. Even if we can’t exclude the persistence of poly(:C) in oyster cells, altogether these results confirmed that poly(I:C) priming is a long-lasting phenomenon, efficient in protecting oysters in the field.

## Discussion

In the present study, we characterized the extent of nucleic acid antiviral priming in the oyster. We demonstrated for the first time that nucleic acids of different structures (single or double stranded) or various lengths (from 300 bp to 8 kb) could all be efficient in inducing an antiviral state and able to reduce mortalities upon viral infection, ranging from 83% to 100% survival. In these trials, poly(I:C) HMW was the most efficient in mitigating oyster mortalities (100% survival). These results are in accordance with studies in vertebrates that provide evidence that the length of dsRNA drives distinct innate responses in different cell lineages and demonstrate that HMW poly(I:C) induces stronger antiviral response than LMW poly(I:C)^34^. If poly(I:C) was drastically reducing viral loads in infected oysters, viral loads and mortality rates did not seem to be clearly related for other nucleic acid tested. That discrepancy might be due to the fact that we did not obtain 100% protection rates for this priming experiment. Previous studies also showed variability in responses to dsRNA injection possibly due to variability in accurate administration, potential excretion or poor cellular uptake^35,36^. The elevated viral loads observed might thus be due to sub-optimal injections (low doses or inefficient injections) and further sampling of infected oysters. In addition, we cannot exclude the hypothesis that oysters might, as in vertebrate systems, possess different pathways to recognize different nucleic acids inducing different antiviral responses and different kinetics of viral countermeasures. In mammals, innate immune sensing of nucleic acids represents the major antiviral defense mechanisms^37^. Nucleic acids and poly (I:C) have been described as effective viral PAMPs recognized by cytosolic or endosomal specific receptors (RIG-I-like receptors, Toll-like receptors, cGAS) as well as various nuclease systems^38–43^. They participate in inducing type I IFN to restrict the propagation of foreign genetic material in ultimately interfering with virus assembly and protein translation, or in the induction of several types of cell death including apoptosis^44,45^. In the oyster, *in silico* analyses of the *C*. *gigas* genome revealed the conservation of numerous nucleic acid receptors and components of the IFN pathway (TLRs, RIG-like receptors, PKR, Interferon Stimulated Genes)^30,46–50^. Moreover, previous studies revealed that poly(I:C) and dsRNA induce genes related to the mammalian IFN pathway^29,31,51–53^. These indications of structural or functional conservations of nucleic acid sensing pathways in the oyster could explain the potent activity of a broad range of nucleic acid^46,48,54^. In the light of recent studies, Crisan *et al*. (2016) have also hypothesized that damage-associated molecular patterns (as nucleic acids) might induce long-term effects on the innate immune system resulting in innate immune memory^55,56^. We could then hypothesize that nucleic acids might have a similar capacity to that observed for certain microbes or ligands in priming the immune responses to remember a previous injury. Still, more studies are needed and are currently undergoing in our laboratory to unravel the molecular and cellular bases behind immune priming and the specificity of different nucleic acids recognition. The identification of the receptors, effectors, and signaling pathways behind these processes might not only contribute to the design of other antiviral strategies for virus control in invertebrates, but might also provide a better understanding of the conservation of the invertebrate innate antiviral system.

Based on the demonstrated efficiency of HMW poly(I:C) antiviral priming, we further used this *in vivo* model of interaction to complete the characterization of the antiviral immune priming in the oyster. The use of poly(I:C) in place of a replicating virus allowed to analyze immune responses in a non-pathogenic context, in the absence of any interference that may occur *via* specific viral proteins.

We originally showed that the viral load reduction (OsHV-1 DNA detection below quantification limits) due to poly(I:C) priming was related to a significant decrease in oyster mortalities when facing the OsHV-1 virus with an average survival rate of 95% (ranging from 100% to 78%) over experiments in laboratory conditions. In addition, we demonstrated for the first time that the priming effect was as efficient in experimental challenges using OsHV-1 as in the field when facing a naturally occurring disease outbreak. Priming with poly(I:C) indeed induced in the field, over two independent deployments during two successive years, an average survival rate of 88% (ranging from 80 to 93%). Once again, if viral loads remained low nine days after immersion in the field, we were able to detect virus DNA in primed oysters at 14 days after immersion, when the mortality rates were still low. This is probably due to heterogeneity in efficiency of poly(I:C) treatment and the fact that we tested a batch of oysters that did not reached 100% survival and experienced from 8% to 17% mortality. In mammals, Poly(I:C) was also shown to be a good therapeutic agent against herpes simplex virus (HSV-2) that did not prevent viral infection but decreased disease^44^. Interestingly, previous studies on the causative agents and etiology of the massive juvenile oyster mortalities revealed that the respective role of bacteria *versus* OsHV-1 was still unclear in this polymicrobial disease hampering development of effective prophylactic and therapeutic interventions^57–59^. Our results originally demonstrate that when the virus replication is impaired, disease does not occur which strengthens the hypothesis that the OsHV-1 virus plays a major role in triggering the disease^19^. However, additional studies will be required to decipher whether oysters actually die from a viral disease or from a secondary bacterial infection as demonstrated for vertebrate viral diseases. In these cases virus would act as a primary offender leading to dysfunctional innate immune response and enhanced susceptibility to bacterial infection^60,61^.

We also investigated the specificity of poly(I:C) priming in the oyster. When challenged with a pathogenic bacterial strain, poly(I:C)-primed oyster survival did not exceed 13%. This result suggests that poly(I:C) would specifically induce an antiviral response. In invertebrates, a variety of studies have addressed the specificity of enhanced protections qualified as immune priming, and observed notable differences, from cross-reactivity (non-specific)^62^ to highly specific^63,64^. The degree of specificity associated with this phenomenon is thus likely to be dependent on the pathogen and immune mechanisms involved. However, in the oyster, previous studies showed that *C*. *gigas* possesses distinct and specific antiviral and antibacterial responses^53^, but also that priming with heat-killed bacteria wasn’t efficient in protecting oysters against OsHV-1^30^. Added to the fact that nucleic acid injection induces IFN-like pathways in vertebrates and invertebrates, not regulated following bacterial infection, it further suggests that the protection might be specific to an antiviral protection^29–31,53^. Nevertheless, future work should test challenging poly(I:C) (or other nucleic acid)- primed oysters with different pathogenic bacterial strains to generalize this statement and confirm whether it is a hallmark of oyster antiviral immune priming.

Furthermore, we showed that poly(I:C) increased survival rates in a long-lasting way. Our results demonstrated for the first time that this protection could last for more than 5 months in laboratory and environmental conditions. In human tissues, poly(I:C) possesses a short half-life (4 hours) and is rapidly degraded likely due to endonuclease activities^24,44,65^. In seawater or in the oyster’s hemolymph, we showed that poly(I:C) persistence did not exceed 2 days^65^. Likewise, Masood *et al*. observed in the oyster *Saccostrea glomerata*, that poly(I:C) detection in the hemocytes decreased significantly from 15 h post-injection, also suggesting a short lifetime in oyster tissues^66^. These data suggest that immune priming may not be due to a persistence of poly (I:C) in oyster tissues. Only a few studies have addressed the time course of innate immune priming and examined resistance at different time points after priming. In those studies, performed in various invertebrates including crustaceans, insects and even cnidarians, the improved response to the different pathogens was often shown to be short-lived and did not exceed 3 months^15,67^. In molluscs, duration was not explored over 56 days for *B*. *glabrata/S*. *mansoni* interactions^16^. In *C*. *gigas*, the duration limit was not explored over 54 h for poly (I:C) stimulation and over 7 days for vibrio immune priming^22,23,30,31,68^. In our case, the duration observed thus largely extended over the one observed in previous experiments using poly(I:C) and might even extend over that period since we did not test longer timespans. To our knowledge this is the first demonstration of such duration in a mollusc or invertebrate species. These results, added to the fact that primed oysters are still able to induce antiviral protection a long time after elimination of poly(I:C), are suggestive of the existence of innate immune memory mechanisms in the oyster. However, the notion of innate immune memory in invertebrate is an intense subject of debate. This notion has been introduced by Kurtz *et al*.^4^ in a phenomenological approach that wasn’t supported by mechanistic analyses. Since then, numerous studies have tried to come up with a general definition of this phenomenon and attributed to invertebrate innate immune memory multiple mechanisms; from the *sensu stricto* recalled response (where immune parameters have to return to a resting state) to sustained responses or even biphasic shifting responses^69^. In our case, we cannot exclude that oysters are experiencing either of these scenarios and further work are granted to find relevant priming markers to help understand the mechanisms involved in this antiviral protection.

Finally, it is interesting to note that our data were obtained on different cohorts of naïve oysters tested at the same age, thus supporting the fact that the observed innate immune priming effect was not due to a specific genetic background. Hence, these processes seem to be a general trait in oysters, even if we cannot exclude natural variations in priming capacities. Taking this trait into account would be of outmost importance in view of the therapeutic potential of innate immune priming. The possible use of immune priming has indeed been a subject of intense research to develop “pseudo-vaccines” to reduce infection in economically important invertebrates, notably in crustaceans to mitigate recurrent viral diseases^15,70,71^. The practical difficulties associated with vaccine administration and potential safety issues that should be considered for food sources underlie the need to further explore this new strategy of pathogen control. Regarding application potential, it is noteworthy that the observed protection by poly(I:C) was not relevant to the route of priming used^11,72,73^. When testing bathing oysters in poly(I:C), we obtained similar protection results as for injection (Supplementary Figure 3S).

In conclusion, in the present study we demonstrate that antiviral immune priming processes are not restricted to arthropods and are also present in Lophotrochozoans, further highlighting the diversity of invertebrate immune processes. This model may provide better understanding of disease mechanisms underlying viral infections and could be used to explore novel antiviral strategies to help mitigate disease threats upon marine bivalves. Numerous questions are still emerging about characteristics of innate immune priming and more work is warranted to try and draw a comprehensive view of all these processes existing across phyla in regard to the diversity of interactions studies and multiplicity of mechanisms involved. To try and understand the nature and origin of these convergent or conserved innovations, a recurrent question is the molecular bases of immune priming and memory. Future studies should endeavour to investigate molecular bases of this phenomenon as well as its ecological and adaptive significance in oyster populations.

## Materials and Methods

### Experimental animals

Experiments have been performed on specific pathogen -free (SPF) (*i*.*e*. free of mortality, negative for OsHV-1 detection and very low *Vibrio sp*. bacteria concentrations^74^) juvenile *C*. *gigas* oysters (or *Magallana gigas*, Thunberg, 1793) (4–8 months old, less than one-year old) from the FINA Ifremer project and from the DECIPHER ANR program (ANR-14-CE19-0023). SPF oysters were produced following standardized protocols under sanitary controlled conditions at the Ifremer research facility of Argenton. These batches of oysters were chosen for their naive and pathogen-free character and were used indifferently, depending on their availability at the time of the experiments, independently of their genetic background. For reproducibility issues, oysters were always tested at comparative ages (4 months old), except for duration and field experiments where priming efficiencies were monitored until 8 months-old.

### Preparation of pathogens or control inoculums

OsHV-1 inoculums (or virus homogenates) were prepared according to^75^ from moribund oysters experimentally infected with OsHV-1 in previous trials and frozen at −80 °C. All inoculums used in the different experiments discussed in this study were prepared from the same batch of infected oysters to ensure reproducibility (except for experiment in supplementary files). Viral DNA loads (OsHV-1 µvar genomes copies.µL^−1^) in inoculums were estimated by qPCR^19^ and indicated for each experiment. Viral inoculums were confirmed to be free of cultivable bacteria by plating 40 µl on LB NaCl agar plates. Control inoculums (control homogenate) were prepared following the same protocol from healthy naïve oysters showing no detectable amount of viral DNA.

Vibrio inoculum was prepared from *Vibrio tasmaniensis* LGP32 strain that was isolated from *C*. *gigas* undergoing a mortality episode^76^. Vibrios were grown under agitation at 20 °C in Zobell medium for 24 h. Cultures of *V*. *tasmaniensis* LGP32 were centrifuged (1000 × g, 10 min, 20 °C) and re-suspended two successive times in sterile seawater to an optical density (OD600) of 1 (1 × 10^9^UFC/ml).

### Priming solutions

For priming experiment, the following nucleic acids were used: poly(I:C) HMW (Invivogen, cat. code:tlrl-pic), poly(I:C) LMW (Invivogen, cat. code:tlrl-picw), double stranded RNA GFP (GFP_ dsRNA), double stranded RNA OsHV-1 (OsHV1_dsRNA), single stranded RNA OsHV-1 (OsHV1_ ssRNA). Poly(I:C) comprises long strands of inosine poly(I) homopolymer annealed to strands of cytidine poly(C) homopolymer. The average size of Poly(I:C) HMW is from 1.5 kb to 8 kb and the average size of Poly(I:C) LMW is from 0.2 kb to 1 kb. Poly(I:C) solutions were prepared following manufacturer instructions and diluted in sterile filtered (0.02 µm) seawater to the desired concentration. Double stranded and single stranded RNAs were synthesized using the Ambion MEGAscript®RNAi kit (cat.#AM1626). To generate transcription template for both strands of the dsRNA, two separate PCRs with a single T7 promoter-containing PCR primer in each were performed. For OsHV-1 dsRNA and ssRNA, a 330 pb fragment targeting the ORF 87 genomic sequence (Genbank AN KF517121) was amplified using primers from^77^ (with the T7 promoter appended to the 5′ end of the primers) using cDNAs synthesized from RNA extracted fromOsHV-1 infected animals. For GFP dsRNA, a 692 bp fragment targeting the green fluorescent protein sequence was amplified using primers GFP-T7-F-TAATACGACTCACTATAGGGAGAGCGAGGAGCTGTTCA and GFP-T7-R-TAATACGACTCACTATAGGGAGAGTCCATGCCGAGAGT using the plasmid pJGFPH as a template.

### Priming and challenge experiments

For each experimental priming and challenge experiment, animals were previously anesthetized in hexahydrate MgCl_2_ (ACROS, cat.# 197530250, 50 g.L^−1^, 100 oysters/liter) according to^78^ for 4 to 10 h. Oysters were injected using 26-gauge needle attached to a multi-dispensing hand pipette, either with 20 µL or 40 µL of the priming solution and 20 µl of the pathogen/control inoculums in the adductor muscle to spread into the circulatory system. Injection of sterile filtered seawater (FSW- in the same proportion used for nucleic acids resuspension) was used as a priming injection control. For all experimentations, a group of oysters were only anesthetized or non-treated as non-injected control. Non-treated oysters refer to oysters that were not primed, nor challenged in controlled conditions and not primed in the field experiment. For short term rearing (<24 h) oysters were replaced in seawater in plastic tanks at room temperature; for long term rearing after priming (>24 h), oysters were maintained at 14 ± 1 °C and fed in a biosecure nursery facility at Ifremer’s Aquaculture Research Facility in Palavas-les-Flots (Laboratoire Aquaculture en Languedoc-Roussillon, LALR, Southern France). All challenge experimentation were performed in 3 replicates with spats in 1 L of seawater in plastic tanks regulated at 20 ± 1 °C. In each tank, spats were separated in two groups; one for mortality monitoring and one for sampling. Sampling consisted in individually removing shells of each oyster with a sterile scalpel blade and snap-froze the whole oyster in liquid nitrogen by pool of three animals. The oysters were stored at −80 °C before grinding to powder and nucleic acid purification. Mortality was followed daily and dead spats were removed from tanks and stored at −80 °C. Survival rate data were analyzed for statistical differences between treatments by log rank test on Kaplan-Meier survival curves. Figure 3 is depicting priming experiments 4 and 5.

#### Priming specificity studies: testing various nucleic acid injections

Different nucleic acid were used (poly(I:C) HMW, poly(I:C) LMW, GFP_dsRNA, OsHV1_dsRNA, OsHV1_ssRNA). For each condition, 270 spats of a susceptible oyster line (F15-Decipher project) were primed after anesthesia (6 h) with 20 µL (5 µg/oyster) different nucleic acids or with filtered seawater as a control. For each priming condition, 24 h after priming, 135 spats were injected with 20 µl of OsHV-1 inoculum (8.9 × 10^5^ copies of DP gene.µL^−1^) and 135 spats injected with control inoculums, excepted for anesthesia and non-treated conditions.

#### Poly(I:C) HMW dose effect

Different doses of poly(I:C) HMW (19 µg, 1.9 µg, 190ng, 19ng and 1.9ng.g^−1^ of oyster) were injected to oysters after anesthesia. For each condition, 360 spats of a susceptible oyster line (F11-Decipher project) were primed with 40 µL of poly(I:C) HMW at different concentrations after anesthesia (5 h) or injected with filtered seawater as a control. For each condition, 24 h after priming, 180 spats were injected with 20 µL of OsHV-1 inoculum (1.1 × 10^6^ copies of DP gene.µL^−1^)and 180 spats injected with a control inoculums, excepted for anesthesia and non-treated conditions.

#### Poly(I:C) specificity of protection against Vibrio and virus

For each condition, 300 spats from the same 01-2016 FINA cohort (Ifremer production)^74^ were injected after anesthesia (10 h) with poly(I:C) HMW (19 µg.g^−1^ of oyster) or filtered seawater as a control. For each priming condition, 24 h after priming, 100 spats were infected with OsHV-1 (5.6 × 10^5^ copies of DP gene.µL^−1^), 100 spats with a control inoculums and 100 spats with *V*. *tasmaniensis* LGP32 (3.5 × 10^6^ UFC.µL^−1^).

#### Duration of the poly(I:C) protection

For each priming condition (Fig. 3), 1200 spats from the same 01-2016 FINA cohort (Ifremer production)^74^ were injected after anesthesia with poly(I:C) HMW(19 µg.g^−1^ of oyster) and 1200 spats with filtered seawater as a control on the same day. At different time post-priming, *i*.*e*. 1, 14, 28, 56 or 126 days after priming, a group of oysters (120 spats) was infected with OsHV-1 (mean: 7.4 × 10^5^ copies of DP gene.µL^−1^) or control inoculums (120 spats) to determine if the poly(I:C) was still protecting from OsHV-1 infection.

#### Efficiency and duration of the poly(I:C) protection in the environment

A first experiment was performed in 2015. SPF oysters used are from the same 8-months old SPF 01-2015 FINA cohort (Ifremer production). Four groups of 100 oysters were anesthetized in MgCl2 and injected with poly(I:C) HMW (10 µg per oyster) or non-treated as a control, 1 day before immersion in the Thau lagoon (43°22′45.1″N 3°34′16.1″E, France).

A second experiment was performed in 2016. SPF oysters from the 01-2016 FINA cohort (Ifremer production) were injected after anesthesia with poly(I:C) (19 µg.g^−1^ of oyster) or filtered seawater as a control, 126, 99, 71 days or 24 h (priming A to D, respectively, see Fig. 3) before deposition in the Thau lagoon (43°22′45.1″N 3°34′16.1″E, France). Each condition was divided in two: one for sampling (72 spats) and one for mortality monitoring (100 spats). In parallel to immersion in the natural environment, for each priming times, laboratory infections with OsHV-1 µvar (2.2 × 10^5^ copies of DP gene.µL^−1^) have been carried out following previous protocol.

#### Poly(I:C) protection through bath treatment

After anesthesia, 20 oysters were exposed to two doses of poly(I:C) HMW (dose 1 was 0.76 µg.mL^−1^ and dose ½ was 0.38 µg.mL^−1^) in 100 mL seawater baths for 2 and a half hours. Each treatment was performed in duplicate. After 2h30 of exposure, oysters were replaced in a 1 L tank with fresh seawater for 24 h. Each oyster group was then anaesthetized and divided in 2. Twenty oysters for each treatment, as well as non-treated oysters as a control, were injected with a viral homogenate containing OsHV-1 (3.6 × 10^7^ copies of DP gene per oyster) and the 20 others with a pathogen free control homogenate (0 copies of DP gene per oyster) and mortalities were monitored daily for 8 days.

### Nucleic acid purification

Oyster pools were homogenised by bead-beading (Retsch, Mixer Mill MM400) with a stainless steel ball bearing and housing that had been pre-chilled with liquid nitrogen. Genomic DNA was purified from homogenised oyster tissues using the Macherey-Nagel Genomic DNA from tissue kit (cat.# 740952.250) following manufacturer’s instruction with an additional step of RNAseA treatment (Macherey-Nagel, cat. # 740505). The quality and quantity of genomic DNA was estimated on Nanodrop 2000 spectrophotometer (Thermoscientific®). Total DNA was resuspended to a final concentration of 20 ng.µl^−1^ prior to quantitative PCR.

### Viral detection and quantification

Detection and quantification of OsHV-1 DNA was performed using quantitative PCR (qPCR). All amplification reactions were performed in duplicate using a Roche LightCycler 480 Real-Time thermocycler (qPHD-Montpellier GenomiX platform, Montpellier University). PCR reaction volumes were 6 µl containing LightCycler 480 SYBR Green I Master mix (Roche), 100 nM of pathogen specific primers and 20 ng of DNA using an initial denaturation (95 °C for 10 min) followed by 40 cycles of denaturation (95 °C, 10 s), hybridization (60 °C, 20 s) and elongation step (72 °C, 25 s). Virus specific primer pairs targeting a region of the OsHV-1 genome, predicted to encode a DNA polymerase catalytic subunit (DP) were chosen as previously described (ORF100, nucleotides 147655–153291 of AY509253): OsHVDPFor- ATTGATGATGTGGATAATCTGTG/ OsHVDPRev- GGTAAATACCATTGGTCTTGTTCC)^79,80^. For absolute quantification, DP amplification products were cloned into the pCR4-Topo vector and replicated in Escherichia coli DH5a (Invitrogen). Plasmids were extracted using the Wizard Plus SV miniprep DNA purification system (Promega) and standard curves of known concentration of plasmid generated according to the Applied Biosystems manual of absolute real-time RT-PCR quantification. Absolute quantification of OsHV-1 genome copies in oyster samples was then estimated by comparing the observed crossing point (Cp) values to known plasmid standards from 10^3^ to 10^9^ copies of DP. The effect of priming on OsHV-1 μvar load was determined using Kruskal-Wallis test (p < 0.05) using the computer software package, GraphPad Prism® v. 6.0. Mann Whitney test was used to compare rank of means if significant differences were found (p < 0.1).

### Immuno-northern-blotting for Poly(I:C) persistence assay

20 µg of poly(I:C) was diluted in 500 µL of filtered seawater (0.02 µm) or filtered hemolymph (0.2 µm extracted from a pool of 13 oysters) or sterile RNase-DNAse free water as a control and incubated at 17 °C. Each condition was sampled at time 0 h, 24 h, 48 h and 4 days. Presence of poly(I:C) in each sample was first monitored by gel electrophoresis on a 1% agarose in Tris-acetate-EDTA 1X gel. 20 µL of each sample (1 µg of poly(I:C)) and 5 µl of ladder (SmartLadder MW-1700-02, Eurogentec) was electrophoresed during 2 hours at 4 °C and 50 V; nucleic acid presence was revealed by GelRed nucleic acid stain (Biotium) for 10 min. Gel image was taken using the Molecular Imager® Gel Doc™ XR system (Bio-Rad) and the Quantity One® Software Version 4.6.3 (Bio-Rad) after 4 seconds of exposition. Presence of poly (I:C) was secondly revealed by immuno-northern-blotting using the J2 antibody (Scicons) specific to dsRNA^28^, based on the “DIG DNA labeling and detection kit” protocol from Roche (cat.No. 11093657910). A total of 20 µL of each sample, 1 µg of *C*.*gigas* genomic DNA and 2 additional lanes deposited with 1 µg of poly(I:C) were deposited on a non-denaturating 1% agarose TAE gel and electrophoresed for 2 h at 50 V and 4 °C. Gel was blotted onto an Hybond-N nylon membrane (GE Healthcare) by semidry electroblotting in 1X TAE buffer for 45 min (appligene vacuum blotter, 50 mbar) before UV crosslinking using the spectrolinker (spectronics corporation- optimal crosslink at 120000 µJ/cm^2^). The membrane was blocked with a blocking solution containing 10% blocking reagent (Roche Diagnostics) in maleic acid buffer (0.1 Maleic acid, 0.15 M NaCl, pH7.5) for 30 min at room temperature (RT) and then cut in two. The first part was incubated with the primary antibody (J2-scicons) diluted at 1:600 in blocking solution for 1 h at RT. The second one with the 2 additional poly(I:C) lanes was not incubated with the J2 primary antibody and then treated the same way as the first membrane. After washing twice with washing buffer (0.1 Maleic acid, 0.15 M NaCl, pH7.5-0.3% (v/v) tween 20) for 15 min at RT, membranes were incubated with appropriate AP-conjugated secondary antibody (Goat anti-mouse, SIGMA#A0162) diluted 1:5000 in blocking solution. After washing, color revelation was performed by incubating the membrane in color substrate solution (2% NBT/BCIP stock solution (Roche Cat. No. 11681451001) diluted in a detection buffer composed of 0.1 M Tris-HCl-0.1 M NaCl, pH 9.5) in the dark. Membranes were scanned to TIFF files using the Ilssemoquentdenous™ Version 10 (Max®) with no contrast modifications.

## Electronic supplementary material

Supplementary information
