# Supplementary material for: Long-lasting antiviral innate immune priming in the Lophotrochozoan Pacific oyster, Crassostrea gigas
Source: Sci Rep. 2017 Oct 13;7:13143. doi: 10.1038/s41598-017-13564-0 (PMC5640609; doi:10.1038/s41598-017-13564-0)

**Supplementary files**

**Long-lasting antiviral innate immune memory in the Lophotrochozoan Pacific oyster, *Crassostrea gigas***

Maxime Lafont^1,2^, Bruno Petton^3^, Agnès Vergnes^1^, Marianna Pauletto^4^, Amélie Segarra^5^, Benjamin Gourbal^2^, and Caroline Montagnani^1^*

**^1^** Ifremer, IHPE, UMR 5244, Univ. Perpignan Via Domitia, CNRS, Univ. Montpellier, F-34095, Montpellier, France

**^2^** Univ. Perpignan Via Domitia, IHPE UMR 5244, CNRS, IFREMER, Univ. Montpellier, F-66860 Perpignan, France

**^3^**Ifremer, LEMAR UMR6539, F-29840 Argenton-en-Landunvez, France

^4^ Department of Comparative Biomedicine and Food Science. University of Padova, Viale dell’Università 16, 35020 Legnaro (PD), Italy

^5^ Univ. Brest Occidentale, LEMAR UMR 6539 CNRS/UBO/IRD/Ifremer, Institut Universitaire Européen de la Mer, F-29280, Plouzané, France

*Corresponding author- [cmontagn@ifremer.fr](mailto:cmontagn@ifremer.fr)

**Supplementary figure 1 S: Poly(I:C) specifically protects against viral infection in a dose dependent manner**

Kaplan–Meier survival curves generated from spats primed by injection with poly(I:C) HMW at different doses (19µg, 1.9µg, 0.19µg, 0.019µg or 0.0019µg per g of oyster) and injected 1 day post-priming with OsHV-1 µvar homogenate (2.3 x 10^7^ copies of DP per oyster). Priming with sterile filtered seawater (FSW) or poly(I:C) (19µg per gram of oyster) before challenge with control inoculums was used as control (lines are hidden behind non-treated control). Mortalities were monitored for each treatment group comprising 45 oysters (15 per tank) for 10 days after infection. Different letters next to the graphed lines indicate statistically significant difference among treatment with a-b corresponding to *p*-value <0.05; b-c to *p*-value <0.01; c-d to *p*-value <0.001; d-e to *p*-value <0.01 (log-rank test, n=45).


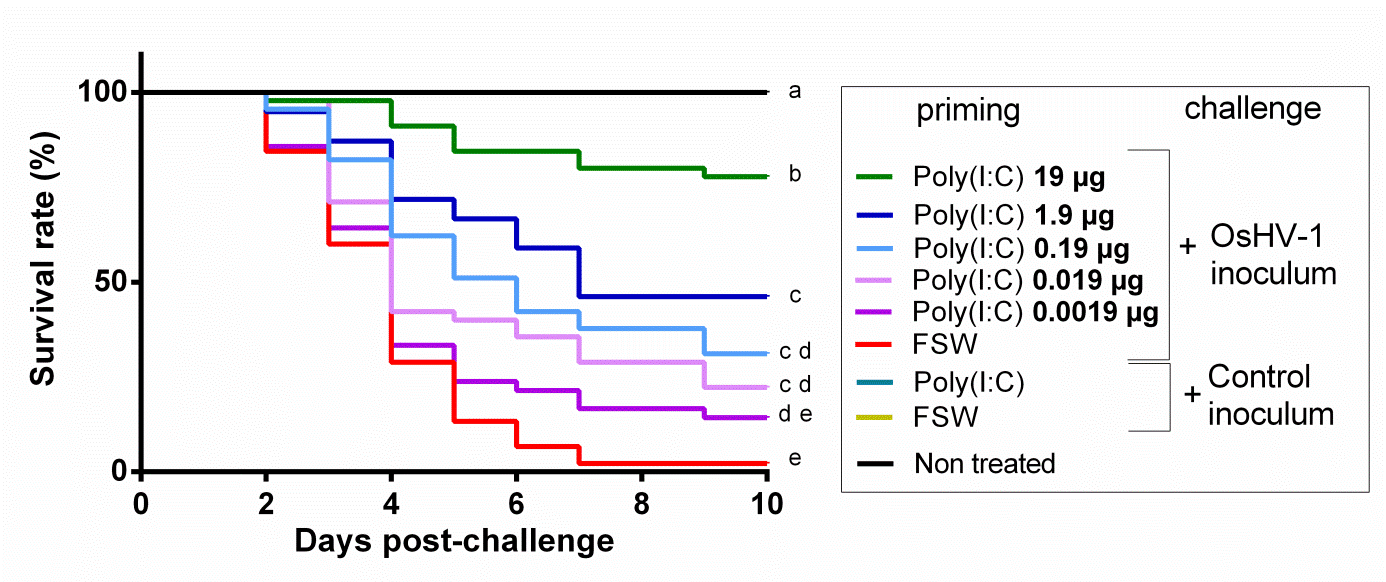


**Supplementary figure 2S: Immuno-northern-blotting for Poly(I:C) persistence assay**


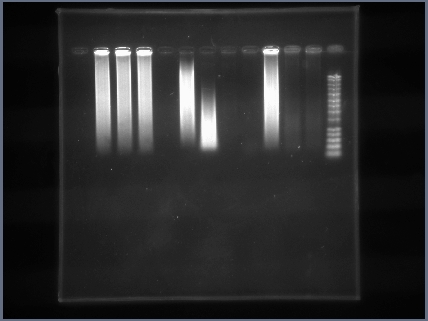
**a.**Uncropped image of Poly(I:C) electrophoresis referencing to Figure 6a. **b.**Uncropped image of poly(I:C) immuno-localized using the J2 antibody (Scicons) referencing to Figure6b.**c.**Uncropped image of control membrane for immune-northern-blotting: 2 lanes loaded with 1µg of poly(I:C) were separated on 1% TAE agarose gel and blotted onto the same membrane. Membrane was cut in two and one part (Figure 6b and supplementary Figure 2sb) was treated with the J2 antibody whereas for the other part (supplementary Figure 2Sc) was only treated with the secondary antibody.

a.

**
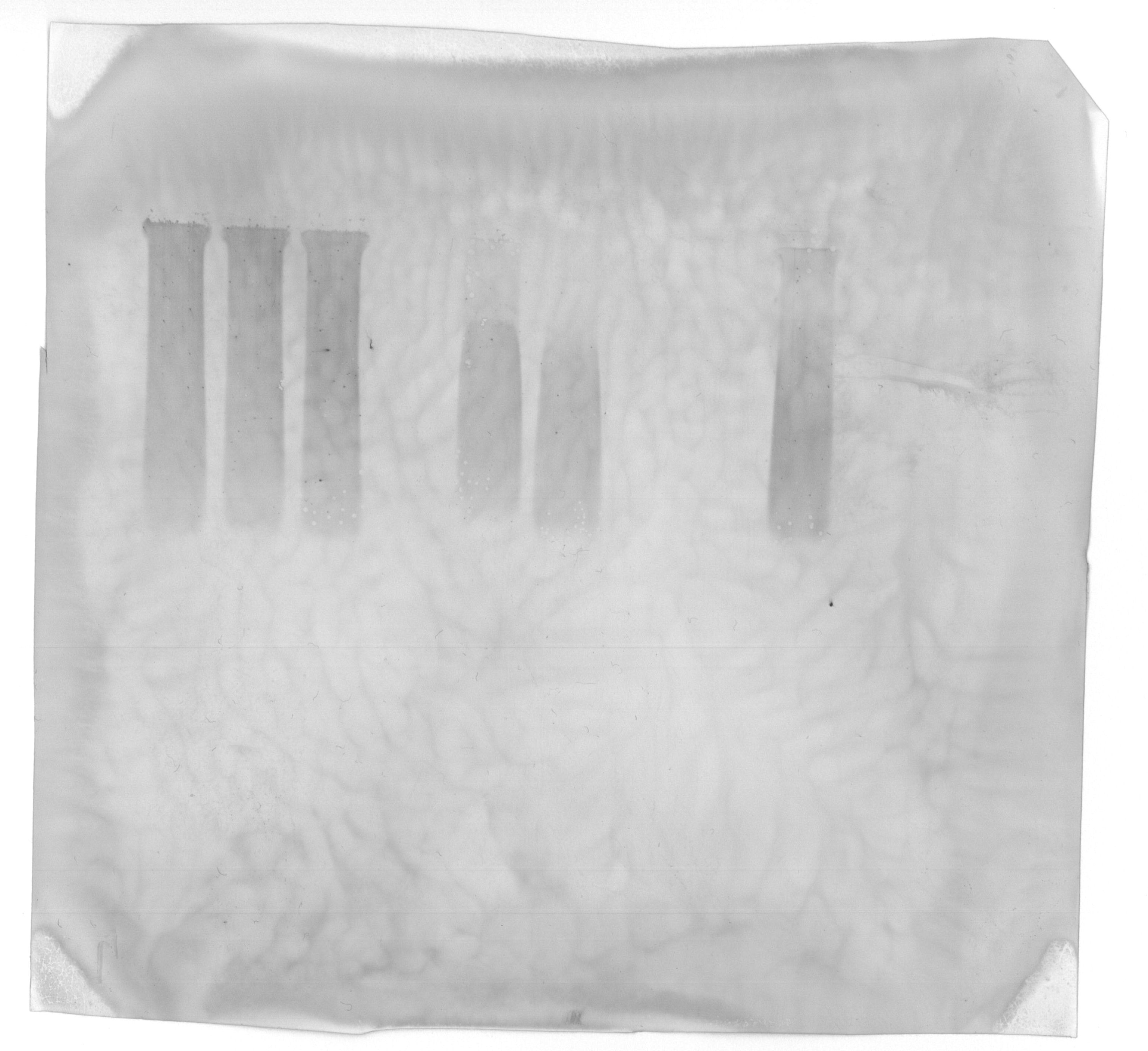
**

b.


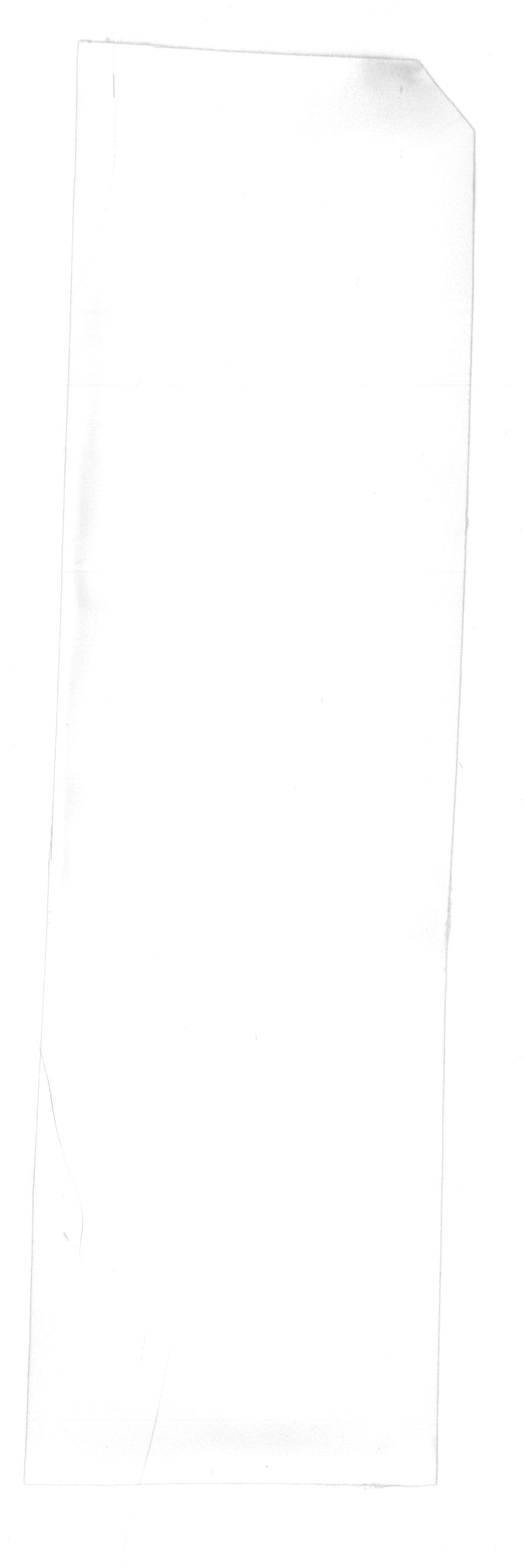
**c.**

**Supplementary figure 3S: Poly(I:C) protection through bath treatment**

Kaplan–Meier survival curves were generated from spats exposed after anesthesia to two different poly(I:C) bath treatments ((1) 76µg.mL^-1^, solid green and blue lines or (1/2) 38µg.mL^-1^, dotted green and blue lines) or only anesthetized as control (red and grey lines) during 2h30. Twenty four hours after treatment, oysters were injected with OsHV-1 homogenate (3.6x10^7^ copies of DP gene per oyster; solid green, dotted green and red line) or with a pathogen free control homogenate (0 copies of DP gene per oyster; solid blue, dotted blue and grey line). Mortalities in groups of 20 oysters per treatment were monitored for 8 days after injection. Different letters next to the graphed lines indicate statistically significant difference among treatment at *p-*value <0.05 (log-rank test;n=20).


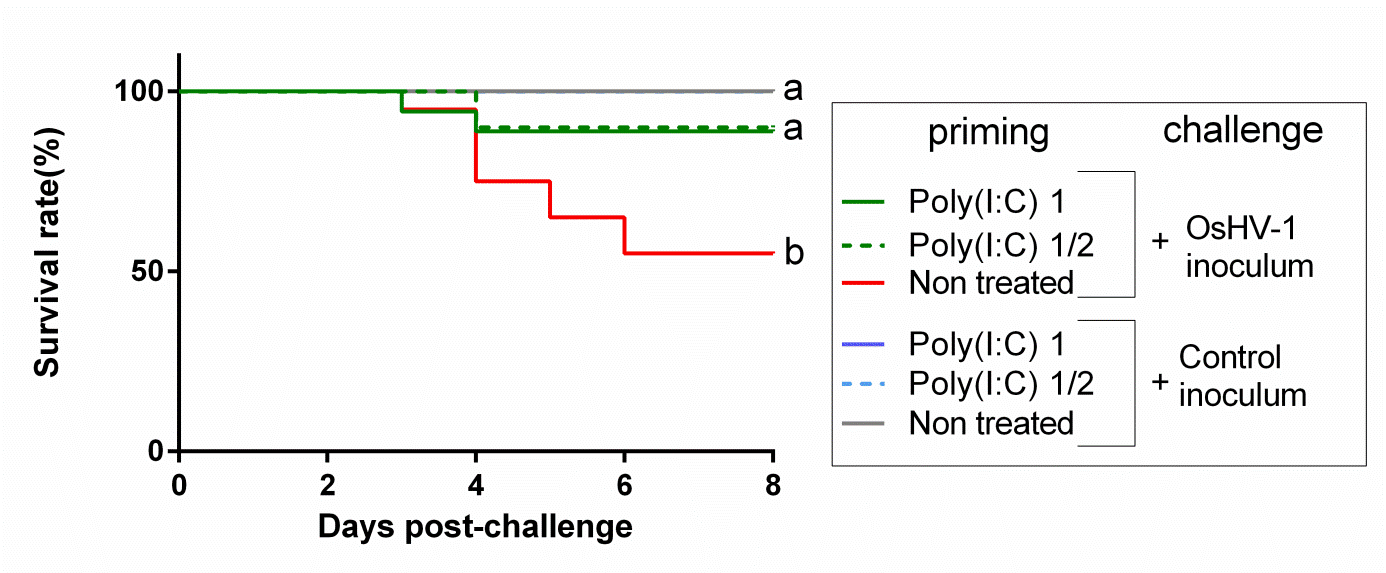

Supplement: Supplementary file 1 — Supplementary information [file 41598_2017_13564_MOESM1_ESM.doc]
